# The Effects of **α**-Lipoic Acid against Testicular Ischemia-Reperfusion Injury in Rats

**DOI:** 10.1100/2012/489248

**Published:** 2012-10-24

**Authors:** Seda Ozbal, Bekir Ugur Ergur, Guven Erbil, Isıl Tekmen, Alper Bagrıyanık, Zahide Cavdar

**Affiliations:** ^1^Department of Histology and Embryology, School of Medicine, Dokuz Eylül University Inciralti, 35340 İzmir, Turkey; ^2^Department of Molecular Medicine, Health Science Institute, School of Medicine, Dokuz Eylül University, 35340 İzmir, Turkey

## Abstract

Testicular torsion is one of the urologic emergencies occurring frequently in neonatal and adolescent period. Testis is sensitive to ischemia-reperfusion injury, and, therefore, ischemia and consecutive reperfusion cause an enhanced formation of reactive oxygen species that result in testicular cell damage and apoptosis. **α**-lipoic acid is a free radical scavenger and a biological antioxidant. It is widely used in the prevention of oxidative stress and cellular damage. We aimed to investigate the protective effect of **α**-lipoic acid on testicular damage in rats subjected to testicular ischemia-reperfusion injury. 35 rats were randomly divided into 5 groups: control, sham operated, ischemia, ischemia-reperfusion, and ischemia-reperfusion +lipoic acid groups, 2 h torsion and 2 h detorsion of the testis were performed. Testicular cell damage was examined by H-E staining. TUNEL and active caspase-3 immunostaining were used to detect germ cell apoptosis. GPx , SOD activity, and MDA levels were evaluated. Histological evaluation showed that **α**-lipoic acid pretreatment reduced testicular cell damage and decreased TUNEL and caspase-3-positive cells. Additionally, **α**-lipoic acid administration decreased the GPx and SOD activity and increased the MDA levels. The present results suggest that LA is a potentially beneficial agent in protecting testicular I/R in rats.

## 1. Introduction

Testicular torsion is a urologic emergency that occurs frequently in the neonatal and adolescent period [1, 2]. It is characterized by a circulatory failure caused by a testis revolving around the vascular peduncle. This condition is most common in infancy and the beginning of adolescence, but it is seen in almost every age group. Nevertheless, 65% of the cases are seen in the pubertal period, more specifically, at the age of 13 [1–4]. In cases with late diagnoses, this condition can result in function loss and infertility in the testis [1, 3].

Torsion of the spermatic cord first leads to a decrease in the blood stream to the testis. In fact, the basic pathology in testicular torsion is ischemia occurring as a result of the torsion, and tissue damage is done by reactive oxygen species as a result of reperfusion [5, 6]. As a result of the reperfusion of the ischemic tissue, toxic-free oxygen radicals, such as nitric oxide (NO^−^), superoxide anions (O2^−^), hydrogen peroxide (H_2_O_2_), and hydroxyl radicals (OH^−^) occur [1, 5, 7].

Moreover, successively occurring cases cause many biochemical and morphological changes in the cells of the testis, which can lead to lipid peroxidation, protein denaturation, DNA damage, and apoptosis [8–10]. Testes are sensitive to free radical damage [5]. Under normal conditions, enzymatic antioxidant defense systems, such as superoxide dismutase (SOD), glutathione peroxidase (GPx), and catalase (CAT), protect those cells from free radical damage. Malondialdehyde (MDA) is an important indicator of lipid peroxidation.

*α*-lipoic acid (LA) is an eight-carbon endogenous cofactor with a disulfide structure. It was described as a strong antioxidant in the 1980s, and it works against oxygen radicals. It is known for catching hydroxyl and nitric oxide radicals, peroxynitrite anions, and hydrogen peroxide and extinguishing single oxygen atoms [11–13]. Both the oxidized (LA) and reduced (DHLA) forms of lipoic acid are capable of scavenging hydroxyl and nitric oxide radicals, peroxynitrite anions, and hydrogen peroxide and extinguishing single oxygen atoms. It is a potent metal chelator, an anti-inflammatory antioxidant, and a modulator of redox-sensitive signaling and a carbonyl scavenger. Furthermore, LA may also act indirectly to maintain cellular antioxidant defense by enhancing the levels of other natural antioxidants, such as glutathione (GSH), tocopherol, and ascorbic acid [12–15].

 In various different studies, it was shown that LA reduces I/R damage in different tissues [16–20]. The histological and immunohistochemical effects of LA in testicular ischemia and reperfusion damage in testis tissue have not been researched until today. The aim of this study is to investigate the protective and antiapoptotic effects of LA against testicular ischemia-reperfusion injury in rats.

## 2. Material and Methods

### 2.1. Animals and Experimental Design

All experiments were performed in accordance with the guidelines provided by the Experimental Animal Laboratory and approved by the Animal Care and Use Committee of the Dokuz Eylul University, School of Medicine. 35 male Wistar-albino rats weighing 180–220 g were used in this study. The animals were maintained on a constant 12 h light/dark cycle at constant room temperature (23 ± 2°C), and humidity (60%) and ad libitum food and tap water throughout the experiments. 

A total of 35 rats were randomly divided into 5 groups: (1) control group (C, *n* = 7), (2) sham group (S, *n* = 7), (3) ischemia group (I, *n* = 7), (4) ischemia-reperfusion group (I/R, *n* = 7), and (5) ischemia-reperfusion + lipoic acid group (I/R + LA group, *n* = 7). 

Surgery was conducted under intraperitoneal injection of pentobarbital (50 mg/kg) anesthesia. All surgical procedures were performed through standard right-sided midscrotal vertical incisions. In sham-operated group, the testes were brought through the incision and then replaced with a fixation to the scrotum. Ischemia was created by rotating the left testis 720° in a clockwise direction for 2 hours. The torsion was maintained by fixing the testis in the scrotum with a 6-0 silk suture. In I/R and I/R + LA groups, following 2 h torsion, 2 h detorsion of the testis was performed [9]. LA (100 mg/kg; 62320 Sigma, Germany) was administered intraperitoneally 30 minutes prior to detorsion [18]. At the end of each experiment, testes tissue samples were obtained for biochemical and histological investigations in all groups.

### 2.2. Biochemical Estimations

All testis tissues were washed two times with cold saline solution and homogenized using a Tissue Lyser (Qiagen, UK) in 50 mM potassium phosphate buffer pH: 7.8, containing 0.5 mmol/L PMSF, 10 *μ*g/mL aprotinin, and centrifuged at 2500 g for MDA analysis.

The MDA assay was based on the condensation of one molecule of malondialdehyde with two molecules of thiobarbituric acid (TBA) in the presence of reduced agents. The TBA + MDA complex was analyzed by HPLC system as described by Tatum et al. [21]. The MDA levels were expressed as *μ*mol/mg protein. 

For the SOD and GPx assay, homogenate was then centrifuged at 11000 ×g for 10 min. The supernatant was used for determination of SOD and GPx enzyme activities.

SOD activity was analysed by colorimetric assay kit (Cayman, MI, USA) and performed according to the manufacturer's instructions. This assay utilizes a tetrazolium salt for the detection of superoxide radicals generated by xanthine oxidase and hypoxanthine. The reaction was monitored at 440 nm using a plate reader (Synergy HT, BioTek Instrument Inc., Winooski, USA). One unit (U) of SOD is defined as the amount of enzyme needed to exhibit 50% dismutation of the superoxide radical. 

The GPx activity was measured by colorimetric assay kit (Randox Laboratories, UK). The enzymatic reaction was initiated by the addition of cumene hydroperoxide (CuOOH) to the reaction mixture containing GSH, NADPH, EDTA, NaNO_3_, and glutathione reductase. The change in the absorbance at 340 nm was monitored. 

Both SOD and GPx activity results were expressed as U/mg protein. The protein content in each tissue was determined using the bicinchoninic acid protein assay (BCA) (Pierce, USA). Bovine serum albumin was used as a standard [22]. All preparation procedures were performed at +4°C. All homogenates were stored at –80°C prior to testing.

### 2.3. Histomorphological Evaluation

All histomorphological analyses described below were performed by two investigators blind to rat's treatment. For histological examination the tissue samples were fixed in 10% formalin in phosphate buffer for 3 days. Afterwards, testis tissues were processed by routine histological methods and embedded in paraffin blocks. Paraffin blocks were placed in rotary microtome (RM 2255, Leica Instruments, Nußloch, Germany), and sections of 5 *μ*m thickness were obtained with disposable metal microtome blades (Type N35, Feather Company, Osaka, Japan). After deparaffinization and rehydration, all sections were stained with hematoxylin-eosine (H-E). 

#### 2.3.1. Examination of Spermatogenesis

Johnsen's score was used to categorize the spermatogenesis on 5 different places in the same histologic section in 20 seminiferous tubules [23]. A score of 0 to 10 was given to each tubule according to epithelial maturation: 10: complete spermatogenesis and perfect tubules; 9: many spermatozoa present and disorganized spermatogenesis; 8: only a few spermatozoa present; 7: no spermatozoa but many spermatids present; 6: only a few spermatids present; 5: no spermatozoa or spermatids but many spermatocytes present; 4: only a few spermatocytes present; 3: only spermatogonia present; 2: no germ cells but only Sertoli cells present; 1: no germ cells and no Sertoli cells present.

#### 2.3.2. Measurement of Seminiferous Tubule Diameter

In each section, the diameters of 10 separate circular seminiferous tubules were randomly measured using a 10x objective. The mean seminiferous tubular diameter MSTD of each testis was determined in micrometers (*μ*m).

#### 2.3.3. Image Analysis Methods

Images were analyzed by using a computer assisted image analyzer system consisting of a microscope (Olympus BX-51, Japan) equipped with a high-resolution video camera (Olympus DP-71, Japan). All sections were digitally photographed. For morphometric evaluation, computerized video camera-based image analysis system (UTHSC Image Tool software version 3.0, University of Texas Health Science Center, San Antonio, TX, USA) was used.

### 2.4. Evaluation of Germ Cell Apoptosis

In order to detect DNA fragmentation in cell nuclei, terminal deoxynucleotidyl transferase-mediated dUTP nick end-labeling (TUNEL) reaction was applied to paraffin sections. DeadEnd Colorimetric TUNEL system kit (In Situ Cell Death Detection Kit, Roche, Manheim, Germany) used for apoptotic cell detection. Serial 5 *μ*m thick paraffin-embedded sections were deparaffinized, rehydrated in graded alcohol, and pretreated in proteinase K (20 *μ*g/mL) for 15 min at 37°C. After washing in phosphate-buffered saline (PBS), specimens were incubated with fluorescein-labeled deoxy-UTP and TdT at 37°C for 60 min. Then, converter POD solution was applied to slides at 37°C for 30 min. Sections were stained with DAB (Roche Diagnostics, Mannheim, Germany) and counterstained with mayer hematoxylin and analyzed by using a light microscope. The apoptotic index was defined as the number of apoptotic TUNEL-positive cells in 20 circular seminiferous tubule cross-sections per testis section. Each section was examined by two persons blind to the treatments and the average was taken [9].

Immunohistochemistry procedure for active caspase-3 (AB3623, Millipore, Temecula, CA, USA, and Polyclonal antibody) was also performed. After deparaffinization and rehydration, sections were then treated with 10 mM citrate buffer (Cat No. AP-9003-125 LabVision) (pH 6) in a microwave oven for 5 minutes. Then sections were washed with PBS and incubated in a solution of 3% H_2_O_2_ for 5 min at room temperature to inhibit endogenous peroxidase activity. After washing with PBS sections were incubated with normal serum blocking solution at 37°C for 30 min. Sections were again incubated in a humid chamber for 18 h at +4°C with antibody active caspase-3 (1/100); thereafter with biotinylated IgG, and then with streptavidin conjugated to horseradish peroxidase at 37°C for 30 min each prepared according to kit instructions (Invitrogen-Plus Broad Spectrum 85-9043). Sections were finally stained with DAB (Roche Diagnostics, Mannheim, Germany) and counter-stained with mayer hematoxylin and analyzed by using a light microscope. 

### 2.5. Statistical Analysis

All data were analyzed by Kruskal-Wallis test using SPSS 15.0 for Windows. Values are presented as mean ± SD. Differences between the two groups were examined with the Mann-Whitney *U*-test. *P* < 0.05 is considered statistically significant.

## 3. Results

### 3.1. Biochemical Analysis

**Figure 1:**
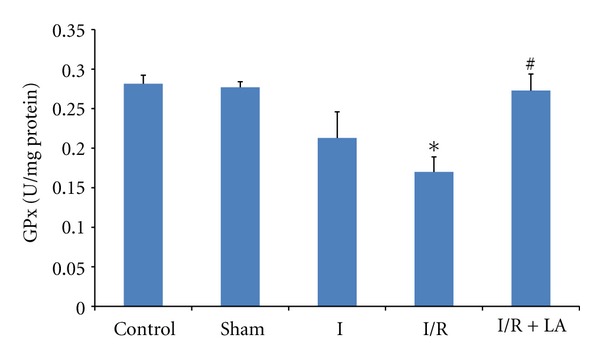
Effects of ischemia/reperfusion (2 h torsion 720° in a clockwise direction and 2 h detorsion of the testis) and LA (100 mg/kg ip, 30 minutes prior to detorsion) on GPx levels of rat testes. Data are mean ± SEM: *P < 0.05 compared with control and sham groups, ^#^ *P* < 0.05 compared with I/R group.

Figure 1 presents the GPx enzyme activities in testis tissue. When we analyzed GPx enzyme activities, we observed that I/R significantly decreased the GPx enzyme activities in the testis compared to control and sham groups (*P* < 0,005 and *P* < 0,003, resp.). However pretreatment with LA increased the GPx enzyme activities as compared to I/R group (*P* = 0,007). There was no significant difference observed between control and sham groups (Figure 1).

**Figure 2:**
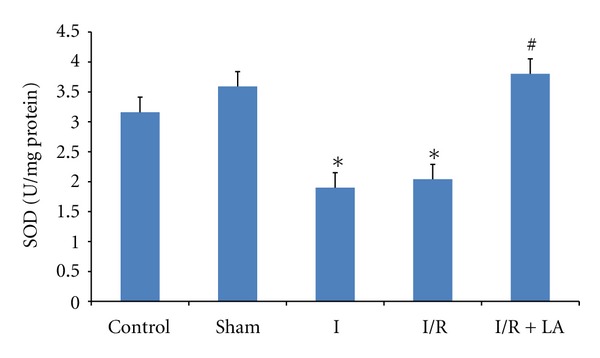
Effects of ischemia/reperfusion (2 h torsion 720° in a clockwise direction and 2 h detorsion of the testis) and LA (100 mg/kg ip, 30 minutes prior to detorsion) on SOD activities of rat testes. Data are mean ± SEM: *P < 0.05 compared with control and sham groups, ^#^ *P* < 0.01 compared with I and I/R groups.

Testis SOD enzyme activities decreased significantly in I and I/R groups when compared to control and sham groups (*P* < 0,05 and *P* < 0,01, resp.). LA pretreatment increased SOD enzyme activities significantly compared to I and I/R groups (*P* < 0,01 and *P* < 0,01, resp.). There was no significant difference observed between control and sham groups (Figure 2).

**Figure 3:**
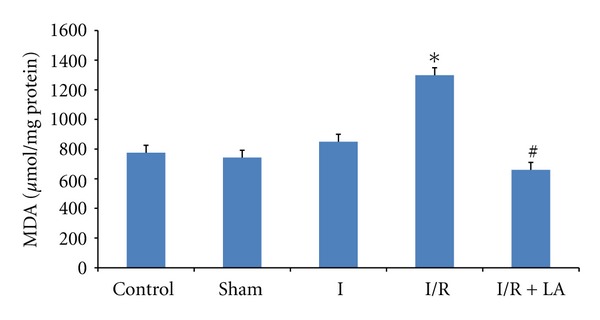
Effects of ischemia/reperfusion (2 h torsion 720° in a clockwise direction and 2 h detorsion of the testis) and LA (100 mg/kg ip, 30 minutes prior to detorsion) on MDA levels of rat testes. Data are mean ± SEM: *P < 0.05 compared with control and sham groups, ^#^ *P* < 0.05 compared with I/R group.

In comparison of MDA levels of testis, we observed that I/R significantly increased the MDA levels in the testis compared to control and sham groups (*P* < 0,028 and *P* < 0,047, resp.). Pretreatment with LA decreased the MDA levels as compared to I/R group (*P* = 0,016). There was no significant difference observed between I and I/IR groups (Figure 3).

### 3.2. Histological Examination

**Figure 4:**
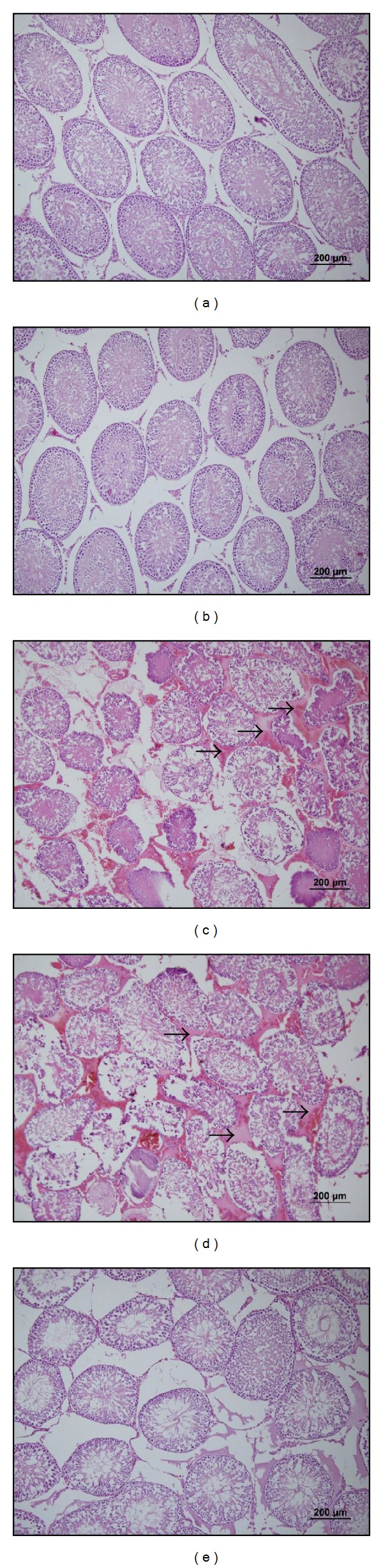
Representative photomicrographs of H-E stained sections of the testis tissue. (a), (b), (c), (d) and (e) show sections of control, sham, ischemia, I/R (2 h torsion 720° in a clockwise direction and 2 h detorsion of the testis) and I/R + LA (100 mg/kg ip, 30 minutes prior to detorsion) groups, respectively. In the I and I/R groups, seminiferous tubular disorganization, degenerative changes and loss of maturation in the germinal cells, interstitial edema, and interstitial hemorrhage were observed. Arrows indicate interstitial edema and hemorrhage.

For histological evaluation, testis sections stained with H-E were examined.  Figure 4 demonstrates the histological findings of each group in testis. In the control and sham groups the animals demonstrated a normal testicular architecture of the seminiferous tubule morphology and interstitium and had intact germinal epithelium (Figures 4(a) and 4(b)). In the I and I/R groups, seminiferous tubular disorganization, degenerative changes and loss of maturation in the germinal cells, interstitial edema, and interstitial hemorrhage were observed (Figures 4(c) and 4(d)). LA pretreated animals showed an improved histological appearance in testes compared with groups I and I/R (Figure 4(e)).

Table 1 compares the parameters in the ischemic testes. In I and I/R groups, there was a marked decrease in the seminiferous tubular diameter and Johnsen's score. MSTD and Johnsen's score decreased significantly in I and I/R groups when compared to control and sham groups (*P* < 0,001 and *P* < 0,001, resp.). On the other hand, LA pretreatment increased MSTD and Johnsen's score significantly compared to I and I/R groups (*P* < 0,001 and *P* < 0,001, resp.) (Table 1).

### 3.3. Effects of LA Pretreatment on Germ Cell Apoptosis

**Figure 5:**
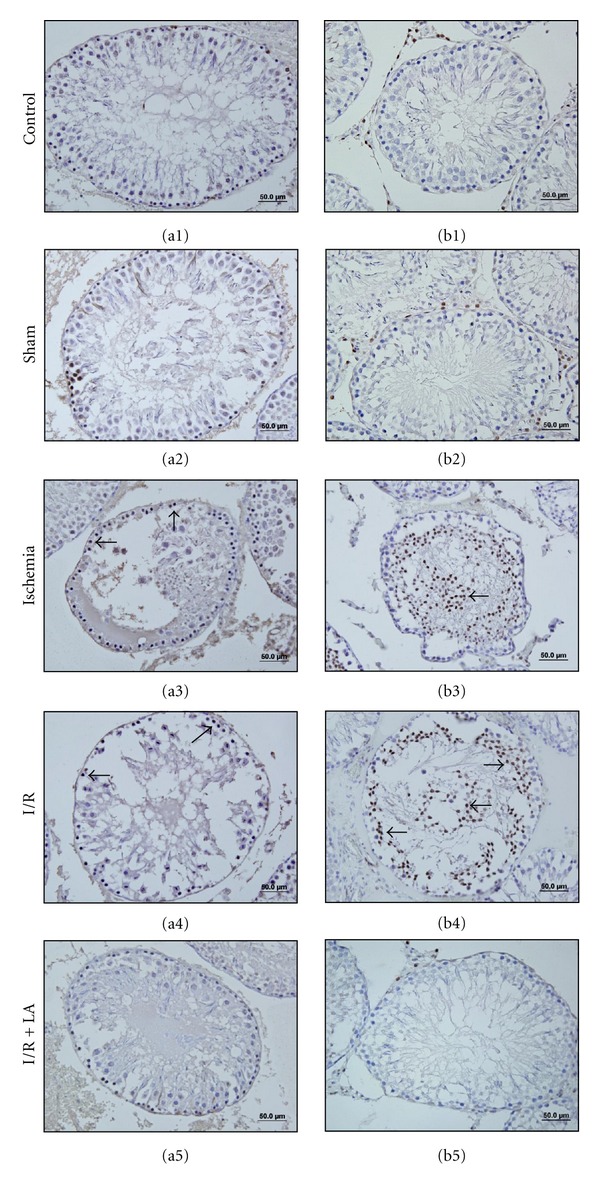
Effects of ischemia/reperfusion and LA (100 mg/kg ip, 30 minutes prior to detorsion) on germ cell apoptosis, TUNEL (a1–5), and active caspase-3 immunoreactivity (b1–5) in the testis tissue. TUNEL-positive and caspase-3-positive cells enhanced in the I (a3–b3) and I/R groups (a4–b4). LA pretreatment significantly reduced the number of apoptotic cells (a5–b5). Arrows indicate TUNEL-positive and caspase-3-positive cells.

The effect of LA pretreatment on germ cell apoptosis was examined by TUNEL assay and active caspase-3 immunohistochemistry. In the I and I/R groups, the cells inclined to undergo apoptosis were distinctively marked (Figure 5). TUNEL-positive cells showed the typical morphological features of apoptosis such as the chromatin condensation, cytoplasmic budding, and apoptotic bodies. Sham and control groups showed fewer TUNEL-positive cells in testis. TUNEL-positive cells were significantly higher in the I and I/R groups compared to control and sham groups (*P* < 0,001 and *P* < 0,001, resp.). LA pretreatment decreased TUNEL-positive cells significantly in I/R + LA group compared with I and I/R groups (*P* < 0,001 and *P* < 0,001, resp.) (Table 1).

Apoptosis was further confirmed by caspase-3 immunohistochemistry. Similarly, sham and control groups showed fewer active caspase-3-positive cells in testis. Active caspase-3 positive cells were enhanced with I/R group when compared with the control and sham group. In the I/R + LA group, however, cells positively stained with active caspase-3 were less observed (Figure 5). 

## 4. Discussion

Testicular torsion is still an important case of male infertility. Mechanisms associated with testicular ischemia, such as free radical generation and lipid peroxidation, are contributing factors. As previously mentioned, ischemia occurring due to the torsion of the testis and the reperfusion related to the detorsioning of the twisted testis can cause various biochemical and morphological changes in the testis tissue. Moreover, reperfusion after ischemia causes an increase in the damage. Therefore, testis ischemia and consecutive reperfusion result in testicular cell damage, and apoptosis.

In order to prevent testicular ischemia-reperfusion injury many were tried. Recently, especially the effects of the substances with antioxidant activities on testicular ischemia-reperfusion injury caused by free radicals have been investigated by many researchers [9, 10, 24–26]. 

LA is a free radical scavenger and a potent biological antioxidant. In humans, it is synthesized in the liver, heart, and kidney [15]. It is a substrate for the Na+-dependent multivitamin transporter, and therefore it not only may contribute to its gastrointestinal uptake, but also may be involved in LA transport into tissues from the blood plasma [12]. LA is absorbed from the diet but also does not extensively accumulate in tissues such as liver, heart, and skeletal muscle but is found in other tissues as well [12, 13]. 20–40% of LA given orally is absorbed into the plasma. Plasma LA levels reach its peak concentration between 0.5 and 2 h after the administration and are rapidly metabolized [13]. Intake of moderate doses of LA has few adverse side effects while long-term LA supplementation and high chronic doses of LA increased plasma lipid hydroperoxide levels and oxidative protein damage [12]. Thus we preferred 100 mg/kg as a single high dose [18].

LA has ROS scavenging capacity, the capacity to regenerate endogenous antioxidants, ability to regenerate endogenous antioxidants such as glutathione, and vitamins E and C, and a metal chelating capacity [14–16]. Unlike other antioxidants, LA is both water and lipid soluble and therefore can cross biological membranes easily and has its antioxidant action both in the cytosol and in the plasma membrane [15].

There are numerous studies in the literature that show the effects of LA against ischemia-reperfusion injury in different tissues and systems. In these studies, it has been shown that LA and its reduced form, dihydrolipoic acid (DHLA), directly scavenge reactive nitrogen species and reactive oxygen species (ROS) by increasing the level of reduced glutathione and by downregulating inflammatory processes. Additionally, as it has also been shown in previous studies, LA also scavenges lipid peroxidation products and induces the enzymes of glutathione synthesis and other antioxidant protective enzymes [16–20]. 

The histological and immunohistochemical effects of LA in testicular ischemia-reperfusion injury in testis tissue have not been researched until today. The aim of the present study is to investigate the possible protective and antiapoptotic effects of LA against testicular I/R injury in rats.

It has been shown by previous studies that testicular ischemia and reperfusion cause histological changes in testis tissue. In the studies carried out with testicular I/R model, degenerative changes in the germinal cells, interstitial edema, and interstitial hemorrhage have been determined in testis tissue [9, 10, 25–27]. Furthermore, it was observed in these studies that there was a decrease in the mean seminiferous tubular diameter (MSTD), germinal epithelial cell thickness (GECT), and mean testicular biopsy score (MTBS) in I and I/R groups [9, 28]. Similarly, testis tissue damages including degenerative changes in the germinal cells, interstitial edema, and interstitial hemorrhage as well as was a decrease in MSTD and Johnsen's score observed in our study. On the other hand, it was observed that LA pretreated animals showed an improved histological appearance in testes compared with groups I and I/R while the MSTD and Johnsen's scores were decreased significantly in the I and I/R groups when compared with that of the control and sham groups. These findings showed that LA pretreatment animals showed an improved histological appearance in testes compared with groups I and I/R. 

Apoptosis is a programmed cell death that occurs in both physiologic and pathologic stages. Many studies have been investigated to examine the antiapoptotic role of LA [29–32]. Our review of the literature did not reveal any studies that investigate the antiapoptotic effects of LA on testis tissue.

TUNEL stains all the cells with fragmented DNA. Since TUNEL stains both necrotic and apoptotic cells, its reliability is insufficient in studies aiming to determine the apoptotic cells alone. Thus, in our study, we preferred to confirm the apoptosis with also active caspase-3 immunostaining. In the present study, the testicular cells inclined to undergo apoptosis were distinctively marked in the sections stained with TUNEL and active caspase-3 in I and I/R groups. TUNEL-positive and active caspase-3 positive cells were lower in the I/R + LA group when compared to I and I/R groups, and it was seen that the pretreatment had a positive effect. This is the first study demonstrates the antiapoptotic effects of LA against testicular ischemia-reperfusion injury in rats. Our study demonstrated that the administration of LA caused a significant decrease in TUNEL and active caspase-3-positive cells on testis tissue.

Recent studies point out that the effects of ischemia-reperfusion injury are associated with the oxidative stress caused by free radicals in tissues. Free radicals interact with polyunsaturated fatty acids in the membrane and start peroxidation [1, 5, 7, 9]. In studies where I/R model is applied, it has been reported that GPx and SOD reactivity was decreased, MDA levels were increased, and the agents that reduce ischemia decrease these levels [9, 33]. Similarly, it was observed in our study that SOD, GPx, and MDA levels in the LA group approached those of the control and sham groups.

In conclusion, it was determined through our study that LA decreased the cellular damage and apoptosis against testicular I/R injury in rats. Additionally, LA administration showed significant protective effects against antioxidant stress by decreasing the GPx, SOD activity, and increasing the MDA levels. These results suggest that LA pretreatment has beneficial effects in the prevention of I/R injury of the testis, and the potency of LA makes it an attractive for the future studies in the protection of testicular I/R injury and candidate for clinical applications. 

## Figures and Tables

**Table 1:** The effect of ischemia-reperfusion (2 h torsion 720° in a clockwise direction and 2 h detorsion of the testis) and LA (100 mg/kg ip, 30 minutes prior to detorsion) on MSTD, Johnsen's score and germ cell apoptosis.

	MSTD (*μ*m)	Johnsen's score	% TUNEL
(1) Control (*n* = 7)	281.03 ± 4.82	9.66 ± 0.05	0.87 ± 0.04
(2) Sham (*n* = 7)	275.37 ± 5.89	9.46 ± 0.7	0.96 ± 0.05
(3) I (*n* = 7)	220.62 ± 2.81*	6.51 ± 0.09*	3.32 ± 0.05*
(4) I/R (*n* = 7)	217.17 ± 3.37*	6.40 ± 0.08*	3.49 ± 0.03*
(5) I/R + LA (*n* = 7)	260.18 ± 2.97**	8.04 ± 0.09**	1.90 ± 0.03**

Values are mean ± SEM.

*P < 0.05 compared with the control and sham group.

**P < 0.05 compared with the I and I/R group.
